# A multistage elastocaloric refrigerator and heat pump with 28 K temperature span

**DOI:** 10.1038/s41598-019-54411-8

**Published:** 2019-12-06

**Authors:** Ryan Snodgrass, David Erickson

**Affiliations:** Sibley School of Mechanical and Aerospace Engineering, Cornell University, Ithaca, NY USA

**Keywords:** Mechanical engineering, Energy science and technology

## Abstract

Elastocaloric refrigerators are a promising alternative to the vapor compression cycle because they do not require refrigerants with high global warming potential. However, these coolers have yet to achieve temperatures low enough to — for example — be used as standard household refrigerators. We built one-stage, two-stage, and three-stage elastocaloric cooling systems to determine if staging of the elastocaloric effect could significantly expand temperature span. Our three-stage system achieved 1.5 times the maximum temperature span of our single-stage system, and produced the highest temperature span of any elastocaloric device to-date at 28.3 °C, where previously the record was 19.9 °C. Interestingly, we found that multistage systems can achieve equivalent temperature spans but at higher coefficients of performance compared to similarly-constructed single-stage systems.

## Introduction

The vapor compression cycle has remained the primary refrigeration method since its invention almost 200 years ago. Although many take reliable refrigeration for granted today, there are many reasons to consider alternative cooling technologies. First, any alternative with improved mechanical efficiency would be of great importance, as an extraordinary amount of energy is spent on cooling every year. About 20% of all electricity used in buildings today is spent on air conditioning, and the global demand for space cooling is projected to triple^1^ by 2050 due to the increasing populations and standards of living in countries with high cooling degree days such as India, Indonesia, and China^2–4^. A second reason to pursue alternatives is the environmental concern of refrigerants leaked into the atmosphere. The 1974 discovery^5^ that chlorofluorocarbons and hydrochlorofluorocarbons were depleting the ozone layer led to the phase-down of those refrigerants, and the high global warming potential^6,7^ (GWP) of today’s refrigerants is leading to their phase-down as well. The use of HFCs (hydrofluorocarbons) is to be dramatically reduced by 2045 according to the ratified Kigali Amendment to the Montreal Protocol^8^.

These concerns have motivated research in more-efficient, lower-GWP refrigerants^9,10^, as well as research in complete alternative methods to the vapor compression cycle^11,12^. One such group of alternatives, caloric cooling methods^13,14^ (magnetocaloric, electrocaloric, barocaloric, and elastocaloric) are attractive because they use solid state refrigerants with no ozone depletion potential or GWP, and have no risks associated with toxicity or flammability. In 2014 the United States Department of Energy released a report concluding that magnetocaloric and elastocaloric systems were two of the most promising alternatives to the vapor compression cycle^15^. Magnetocaloric cooling is the most mature of any of the caloric techniques: many room temperature systems have been built^16^, and adiabatic demagnetization refrigerators use the magnetocaloric effect to achieve cooling at temperatures <1 K^17^. Elastocaloric cooling — although less-studied than magnetocaloric — is particularly attractive because it has proven effective in producing large temperature spans (*T*_span_, the difference between hot and cold outputs) at high coefficients of performance (COP)^18^. Elastocaloric cooling is achieved using shape memory alloys such as Nitinol (NiTi), a metal which is easily accessible because it is used in a variety of other applications such as medical implants^19^. To achieve cooling, elastocaloric devices first induce a crystal structure phase transition in a shape memory alloy by mechanically loading it. The alloy changes from the low-stress state (austenite) to the high-stress one (martensite), and the alloy warms. This heat is then removed and the alloy is unloaded, causing the reverse phase transition and subsequently causing the alloy to cool. The thermodynamics of shape memory alloys is described more completely in the literature^20–24^.

There have been a variety of numerical^25,26^ and experimental attempts at elastocaloric cooling and heat pumping in the past decade. Some devices have achieved heat exchange between a sink and source by direct contact with the refrigerant. These conductive systems have achieved *T*_span_ between 7 and 13 °C^27–29^. Other experimental attempts have used fluids to transfer heat. A rotary system with many NiTi wires used air for heat exchange, generating a *T*_span_ of 10 °C^30^. The highest *T*_span_ to-date was produced by a regenerative device that uses water as heat transfer medium, and that operates similarly to active magnetocaloric regenerators^31^. This device achieved up to a 15.3 °C *T*_span_ at a COP of 3.5^18^, and up to a 19.9 °C *T*_span_ under no-load cooling conditions^32^. Although these temperature spans are impressive, a major concern is that they are still much less than what can be achieved using the vapor compression cycle. The regenerative device only reached about 5 °C below ambient when operating as a heat pump^18^, and the coldest temperature ever achieved in any elastocaloric device (to the best of our knowledge) is 7 °C below ambient^28^ — insufficient to be used in many refrigeration applications.

An increased *T*_span_ is perhaps the most-needed development for elastocaloric cooling devices^33^. The goal of our study was to determine if staging (i.e. multiple refrigeration steps) could significantly increase the *T*_span_ of an elastocaloric refrigerator and/or heat pump. To the best of our knowledge, the only previous work on multistage elastocaloric coolers is a three-stage device using Ni-Ti-Cu-Co films^34^ that produced a *T*_span_ of 15 °C — lower than that achieved using a regenerative approach^18,32^. We built a multistage elastocaloric cooler using NiTi wires and performed experiments in single-stage, two-stage, and three-stage refrigeration configurations, obtaining a minimum temperature of 12.3 °C below ambient and overall maximum *T*_span_ of 28.3 °C – by far the greatest in any elastocaloric system to-date. We also identified certain scenarios where using a multistage approach produced higher COPs than a single-stage approach for the same *T*_span_.

## Results

### System design

**Figure 1:**
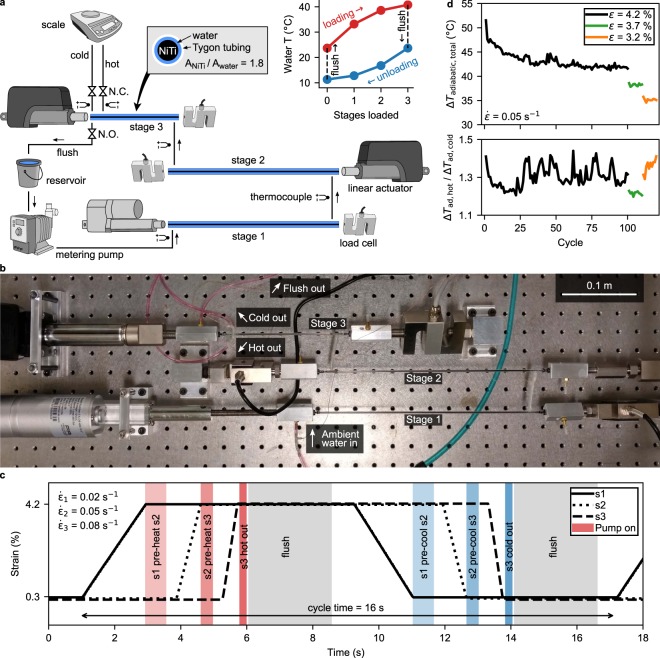
Multistage refrigerator overview. (**a**) Schematic for the three-stage configuration. Linear actuators are used to strain the refrigerant. Stages 1 and 2 are made with NiTi wires roughly twice as long as in stage 3. A metering pump is used to pump water through the system after loading or unloading. A solenoid valve is normally open (N.O.) back to the water reservoir, but when collecting hot or cold water the valves switch function and the normally closed (N.C.) valves allow water to flow to an analytical scale, which measures the mass of water heated or cooled. Top-middle inset shows a cross-section of the refrigerant. Top-right inset shows the maximum (during loading) or minimum (during unloading) water temperature in the system. (**b**) Image of the same system in the same orientation. (**c**) A single cycle for the three-stage configuration showing the timing of the actuators and pump. Stage 1 abbreviated s1. The period (16 s) is dominated by flush steps and the movement of the actuators, especially in the first stage. The stage 1 actuator is about half the speed of the stage 2 and 3 actuators due only to equipment availability. A roughly 100 ms delay gives the solenoids time to change state between the hot/cold out and flush steps. (**d**) (Top) The total (hot and cold) adiabatic temperature change of an as-received NiTi wire (high-*A*_f_) for 100 cycles at 4.2% strain, followed by 10 cycles at 3.7% strain, and 10 cycles at 3.2% strain. (Bottom) The adiabatic temperature change during loading normalized by the adiabatic temperature change during unloading. Adiabatic measurements were performed in air: the wires were returned to *T*_amb_ by heat exchange with ambient air for three minutes between half-cycles.

A schematic of the three-stage elastocaloric refrigerator (which also functions as a heat pump, but we will refer to it as a refrigerator for short) can be seen in Fig. 1a, and a photo in the same orientation in Fig. 1b. The refrigerant in each stage is a 1.27 mm diameter NiTi wire fixed between a linear actuator and load cell. The NiTi wires were placed inside slightly larger, flexible Tygon tubing (1.59 mm inner diameter), creating a small region for water to flow over the wires for heat exchange. Figure S1 shows details for how the wires are fixed and the methods for fluid flow around the NiTi.

Martensitic phase transformation was induced by straining the wires, and hot water was extracted from the system by turning on a metering pump nearly immediately following transformation. After hot water extraction, an additional, larger flow volume was used to bring the wires back to the ambient room temperature (*T*_amb_) from their semi-hot state (this step hereafter referred to as the flush). Then the wires were released and the same pattern of flows were used to extract cold water from the system and then return the refrigerant to *T*_amb_. In multistage operation, the wires were loaded or unloaded sequentially with small bursts of fluid flow in-between for pre-heating and pre-cooling of subsequent stages (Fig. 1c). At the exit of the final stage we placed a series of valves to direct the water into cold, hot, or flush streams. The volume of cold and hot water collected was measured using analytical scales, and temperature via thermocouples placed directly in the water (see Methods and Fig. S2 for details).

### Five staging configurations

We tested our system in three-stage, two-stage, and single-stage configurations all using a NiTi alloy with austenic finish temperature (*A*_f_) near 22 °C. When strained to 4.2% at a strain-rate of 0.05 s^−1^ we measured the adiabatic temperature change (Δ*T*_ad_) of this wire to be about +24 °C during loading and −18 °C during unloading (Figs. 1d and S3), which matches very closely to the Δ*T*_ad_ reported in literature^35^. Under the hypothesis that a NiTi alloy with lower transition temperatures would be required to maintain cooling capacity at the third (and most pre-cooled) stage, we also performed testing of the three-stage system with a lower transition temperature wire (*A*_f_ ≈ 16 °C) placed at only the third stage (henceforth we will refer to this material as the low-*A*_f_ wire). Transition temperatures and latent heats (Δ*H*) were measured via differential scanning calorimetry (Fig. S4). Except for the three-stage configuration with low-*A*_f_ wire at the final stage, all other configurations and experiments exclusively used the higher transition temperature (high-*A*_f_) wire, as that alloy produced larger temperature changes due to a larger Δ*H* (Δ*H* and transformation temperatures have shown to be directly correlated^36,37^). Since the addition of new stages adds considerably to the overall system complexity, we also tried to combine two pre-cooling/pre-heating stages into one by expanding the number of NiTi wires in a single-stage from one to four. Compared to the three-stage configuration, this 4-wire stage has the same overall refrigerant mass dedicated to pre-cooling. In total, we tested five different staging configurations: three configurations only comparing the number of stages, one configuration testing a low-*A*_f_ wire at the most-pre-cooled stage, and one configuration that attempted to combine two pre-cooling stages into one.

### Optimization for maximum *T*_span_

The performance of a multistage system is dependent upon the individual stages and the coupling between those stages. Before presenting the results of our multistage refrigerator, we here discuss many of the parameters important for overall system performance, where our overarching goal was to achieve the largest *T*_span_.

**Figure 2:**
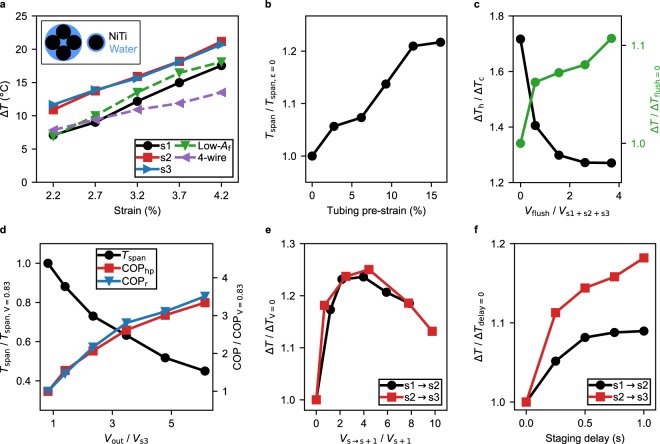
System parameters affecting refrigerator temperature. (**a**) Difference between the maximum and minimum temperature of water leaving the system, Δ*T*, versus strain for each stage (stages tested independently). The inset shows a cross-section comparison for the single-wire stages and the four-wire stage (for all stages *A*_NiTi_/*A*_water_ = 1.8). Stage 1 abbreviated s1. Low-*A*_f_ refers to a NiTi wire with the same dimensions as in stage 3 but with lower austenic finish temperature. (**b**) Difference between the average water temperature leaving the hot and cold streams (*T*_span_) when the Tygon tubing was pre-strained, effectively decreasing the tubing inner diameter. *T*_span_ is normalized by the case when tubing pre-strain is zero. Single-stage experiments. (**c**) The ratio of the maximum temperature change after loading (Δ*T*_h_) to the maximum temperature change after unloading (Δ*T*_c_) against the amount of water used to flush the system back to *T*_amb_. Flush volume is normalized by the volume of water surrounding the refrigerant at all stages. Total Δ*T* is shown and normalized by the case where the flush step is skipped. Three-stage experiments. (**d**) *T*_span_ and COP when the volume of hot or cold water leaving the system, *V*_out_, is varied. *V*_out_ is normalized by the volume of water surrounding the refrigerant (*V*_s3_). *T*_span_ and COP are normalized by their respective values when *V*_out_ was slightly less than *V*_s3_ (*T*_span_ should be maximized when *V*_out_ ≈ *V*_s3_). Single-stage experiment. (**e**) Δ*T* when varying the pre-heating and pre-cooling volume from the previous stage, normalized by the case with no pre-heating/pre-cooling. Pre-cooling volume normalized by the volume of the stage being pre-cooled. Two-stage experiments. (**f**) Δ*T* when varying the delay between stages, normalized by the case with no delay. Longer delays were not tested so that cycle time would remain reasonable. Two-stage experiments.

First we performed baseline temperature measurements of individual stages at a variety of strains up to 4.2% (Fig. 2a). When operated independently, the wires at stage 2 and stage 3 produced nearly identical temperatures. The wire at stage 1 did not produce as extreme temperatures as the later stages, likely because its strain-rate was 2.5 to 4 times lower (due to equipment availability, see Methods or Fig. 1c). The 4-wire pre-cooling stage produced the least-extreme temperatures of any configuration, likely due to the fluid flow around the wires. While the 4-wire stage shares the same volume ratio of NiTi to water as the single-wire stages, the water is not forced to flow as closely to the wires (Fig. 2a inset), resulting in inferior heat exchange. The low-*A*_f_ wire also did not achieve temperature changes as extreme as the high-*A*_f_ wire (both were tested at stage 3), likely because its Δ*H* is less (only 70% as much, see Fig. S4).

The design of our single-wire stages placed a 1.27 mm NiTi wire inside a tube of inner diameter 1.59 mm. We attempted to further decrease the tubing inner diameter by stretching it even when the NiTi was unloaded, with the hypothesis that the Δ*H* could be focused to a smaller volume of water, creating larger temperature changes. When the tubing was pre-stretched to about 16% of its original length, we observed roughly a 20% increase in the maximum temperature change of the water compared to the unstretched tubing (Fig. 2b).

Returning the refrigerant to *T*_amb_ by means of a flush is required before transitioning between loading and unloading. We observed that about 10% of the overall temperature differential can be lost if the flush volume is inadequate (Fig. 2c). An adequate flush volume is especially important when operating the system as a refrigerator, as insufficient flush after loading will cause the Δ*H* during unloading to be wasted on cooling the wire back to *T*_amb_ (Fig. 2c).

Perhaps the parameter with the strongest influence on *T*_span_ and COP is the the volume of water collected for heat pumping or refrigeration: *V*_out_. The highest *T*_span_ can be achieved when collecting small amounts of water from the system (on the order of the volume surrounding the wire), while higher COP is achieved when collecting higher volumes of water (Fig. 2d). This is intuitive, as more heat is transferred to or away from the refrigerant with increasing *V*_out_, albeit at more moderate temperatures. We note that the performance of the refrigerator could be increased by collecting water at multiple outlet temperatures. For example, one methodology would be to first collect the volume of water surrounding the refrigerant (the coldest water), and then separately collect the flush volume to perform cooling at a temperature closer to ambient. This would improve COP in applications where cooling at multiple temperatures can be utilized.

The volume of water used for pre-cooling also affects the performance of the multistage refrigerator. There exists an optimal amount of water to pump from earlier to later stages, which for our system was roughly four times the volume of the stage being pre-cooled (Fig. 2e). We should note that we did not try to completely cool subsequent stages to the maximum temperature change of the previous stage. Such a methodology would require the mass of earlier stages to be much greater than later stages (allowing *V*_out_ to be small relative to the current stage, but high relative to the next). The resulting system COP would be quite low because only a fraction of the cooling capacity would be extracted from the system, with most spent on pre-cooling. In our design, the length (and mass) of the first and second stages were equivalent and only about twice as much as the third stage, producing a result that compromised between overall *T*_span_ and COP.

The high pressure differential required to support the restricted flow around the refrigerant caused the flexible Tygon tubing to slightly expand during pumping, resulting in a flow that did not immediately stop when the pump turned off. We therefore delayed the unloading of the second and third stages after pumping (Fig. 2f) as the maximum temperature change is observed when Δ*H* is concentrated to the smallest volume of water.

### Multistage versus single-stage performance

**Figure 3:**
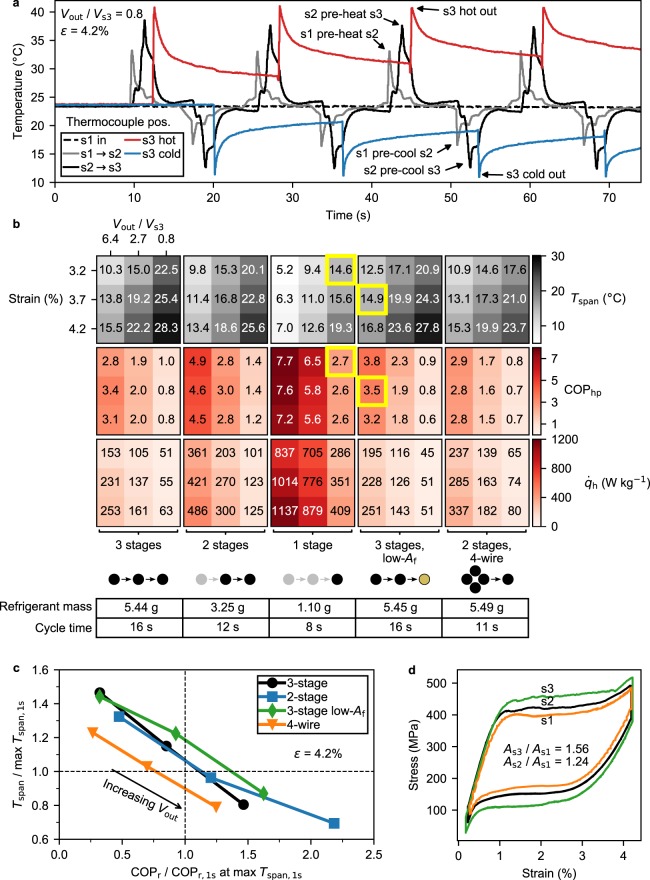
Multistage refrigerator performance. (**a**) The temperature of the three-stage refrigerator during four loading-unloading cycles. See Fig. 1a for the location of the five thermocouples. (**b**) Heatmaps giving heat pump performance for the five different staging configurations (refrigerator heatmaps in Fig. S5). Staging configurations are separated by column, with each respective cycle time and total refrigerant mass below the heatmaps. Each nine-cell square shares the same strain and *V*_out_ layout as the top-left square, and each value in the heatmap is the average of three cycles. *V*_out_ is normalized by the volume of water surrounding the refrigerant in the last stage (*V*_s3_), as maximum *T*_span_ should be observed when *V*_out_ ≈ *V*_s3_. The yellow boxes show a case where the three-stage configuration gives equivalent *T*_span_ but higher COP than the single-stage configuration. (**c**) Multistage performance when normalized by single-stage performance at max *T*_span_ (19.3 °C, corresponding COP_r_ = 1.6: see Fig. S5). (**d**) Stress-strain curves for the different stages during a three-stage experiment (wires with *A*_f_ ≈ 22 °C). The ratio of the areas enclosed by the stress-strain curves is also shown. Nearly-complete reverse superelasticity is observed even in the third stage, likely because the temperature of the wire in that stage does not reach the temperature of the water pre-cooling it (the total heat capacity of the NiTi is roughly 1.5 times that of the water surrounding it).

We chose the system parameters for largest *T*_span_ (i.e. pre-stretched tubing, high flush volume, etc.) and held them constant for all multistage experiments, striving to accurately compare the performance of the five different staging configurations. Real-time operation of the refrigerator can be seen in Movie S1, and Fig. 3a plots data from the three-stage refrigerator, where water temperature decreases progressively with the unloading of each stage. We would like to emphasize that the temperature change in the second and third stages is less than the temperature change from the first stage. This is attributed to the fact that the wires in later stages are not completely pre-cooled to the temperature of the water leaving the previous stage.

We tested a variety of multistage configurations, using single-stage measurements as a control. Because the COP and *T*_span_ of the system depends so heavily on the volume of water collected and the strain applied to the NiTi wires, we tested each of the five staging configurations at nine different *V*_out_-strain combinations (Fig. 3b). We calculated COP using methodology similar to that previously used^18^, under the assumption that only the work taken to stress the refrigerant is significant, and that work-recovery is possible (see Methods for details). *T*_span_ was as high as 28.3 °C (+16.9/−11.4) at a specific cooling power of 42 W kg^−1^ (Fig. S5) using the three-stage configuration with all high-*A*_f_ wires. For the three-stage configuration with the low-*A*_f_ wire in the third stage, a similar maximum *T*_span_ was measured at 27.8 °C, and more of that *T*_span_ was on the cold side (+15.5/−12.3, see Figs. S5 and S6). That the cold-side temperature drop was larger demonstrates that low-*A*_f_ alloys may have an advantage in staged refrigeration applications (note: not in heat pump applications), even though their Δ*H* is generally less^36,37^. The single-wire configuration also achieved an impressive *T*_span_ at up to 19.3 °C. We attribute this large *T*_span_ without staging or regeneration to the small amount of water surrounding the NiTi (see Fig. 1a). When functioning as a heat pump, the system’s COP ranged from 7.7 (single-stage) to 0.6 (three-stage), and when functioning as a refrigerator the COP was slightly lower (Fig. S5), ranging from 6.0 (single-stage) to 0.4 (two-stage with 4-wire pre-cooling). The COP as a refrigerator (COP_r_) is slightly lower because the temperature change of the cold water is less than that of the hot water (Fig. S5) due to hysteresis and temperature irreversibilities^23,24^. COP relative to Carnot was as high as 23% (Fig. S5). In general, *T*_span_ was maximized in higher-stage configurations at higher strains and lower *V*_out_. COP followed the opposite trend.

The expanded *T*_span_ of a multistage system enables some of that *T*_span_ to be traded for improved COP. Figure 3b shows (see the boxes highlighted yellow) that the single-stage system achieved a 14.6 °C *T*_span_ at a COP_hp_ of 2.7, while the three-stage system with low-*A*_f_ wire achieves nearly the same *T*_span_ (14.9 °C) at a much higher COP_hp_ of 3.5. This increase in COP is possible because the three-stage system achieves that *T*_span_ at a much higher *V*_out_ (about eight times more water is collected while only about five times more refrigerant is strained). We only observed this increase in COP when *V*_out_ from the multistage system was quite large compared to *V*_out_ of the single-stage system (eight times greater). An interesting question logically follows: if the single-stage system were built with a wire five times as long (to match the refrigerant mass of the three-stage system), what *T*_span_ and COP would result? While experimentally verifying this question would require an actuator five times faster than we have available, we find it likely that the same performance would result, as *T*_span_ is insensitive to wire length for a single-stage system (Fig. 2a shows stage 2 and stage 3 wires at the same temperature difference, although stage 3 is half the length). This seems to suggest that the increased COP at the same *T*_span_ is not an effect of the increased amount of refrigerant in the multistage configurations, but indeed a result of running the multistage system at a *T*_span_ much less than its maximum.

We directly compared multistage performance to single-stage performance at its maximum *T*_span_. Compared to the single-stage system, the three-stage configurations achieved almost 1.5 times the maximum *T*_span_ while COP_r_ dropped by a factor of three (Fig. 3c). We also observed the divergence of upper and lower transformation plateaus with the addition of each stage (Fig. 3d). If more stages were added to our system and the lower stress plateau were to continue to drop, the NiTi wire may no longer display superelasticity during unloading, at which point it would provide no additional cooling and a wire with lower *A*_f_ would be required (see refs. ^22,38^ for more information). An ideal multistage refrigerator would be built with different alloys at every stage so that Δ*H* is always maximized for but so that pre-cooling does not exceed beyond *A*_f_.

## Conclusions

Staging is a viable way to extend the temperature range of elastocaloric coolers. We achieved *T*_span_ up to 28.3 °C and cold-side temperature changes 12.3 °C below *T*_amb_ (Fig. S5), where previously the records were 19.9 °C and 7 °C, respectively^28,32^. We have also shown that a multistage system can achieve significantly higher COP than a single-stage system at the same *T*_span_. This increased COP is achieved by collecting relatively large amounts of heat transfer fluid and operating the multistage system at a moderate *T*_span_ relative to its maximum.

Possibly the two greatest challenges to the viability of elastocaloric cooling are large *T*_span_ and material fatigue. Fatigue is likely to be addressed by advances in materials: for example, shape memory alloys with lifetimes greater than 10 million cycles have been developed^39^. With the *T*_span_ demonstrated here elastocaloric cooling could realistically be used in air conditioning applications, and it is likely that future systems will reach temperatures cold enough for use as mainstream refrigerators (i.e. for food preservation). Future coolers may increase *T*_span_ further by combining both regenerative^18^ and staged approaches, by increasing the relative mass of refrigerant used in pre-cooling stages, or by increasing the number of stages beyond three. Although our system used three separate actuators, equivalent results could be obtained more simply using just one actuator. For example, a single rotary actuator could drive a spinning system^30^ that would sequentially load an arbitrary number of elastocaloric stages.

## Methods

### System hardware

Products and companies named are noted only for completeness of description and do not constitute endorsement by Cornell University or the authors: other products may be used to achieve similar results. Two Electrak HD linear actuators from Thomson (max speed: 18 mm/s, position feedback resolution: 0.07 mm) were used to strain the NiTi wires in the second and third stage. A Firgelli Automations linear actuator (max speed: 7.5 mm/s, position feedback resolution: 0.25 mm) was used to strain the NiTi wire in the first stage. Strain was measured using the position feedback of the actuators, and therefore is an average strain measurement. We used a slower actuator in the first stage only because a third Electrak HD actuator was unavailable to us, and we believe slightly higher *T*_span_ would have been achieved with relatively fast actuators in all three stages. We always loaded NiTi at the maximum speed of the actuators (although given speeds are maximum, see Fig. 1c for actual strain-rates). Load was monitored using one LC101-2.5 K load cell from Omega Engineering and two TAS-501 load cells from HT Sensor Technology Co (which were calibrated using the Omega Engineering load cell). NiTi wires were fixed between the load cells and actuators using parts designed in-house, see Fig. S1 for details. A metering pump (pressure: 720 kPa, flow-rate: 63 mL/min) from Iwaki was used to flow water through the system. A Teensy 3.5 microcontroller was used to control the actuators, pump, and valves in the refrigerator using an in-house program. All measurement sensors were coupled to the microcontroller. Signals from the load cells were digitized at a sampling rate of about 1 kHz. An acrylic safety enclosure was built around the system in case of wire failure.

### Temperature/volume measurement and COP calculation

The temperature of the water leaving the refrigerator was measured using shielded K-type thermocouples with wire diameter 0.13 mm. MAX31856 thermocouple amplifiers were used (resolution: 0.008 °C, accuracy: ±0.7 °C). Thermocouples were placed directly in the water stream using a tee connector sealed with epoxy. *T*_span_ was calculated using the average temperature during water flow (see Fig. S2a for details). The volume of water flowing through the system was measured using two analytical balances from Denver Instrument with resolution of at least 1 mg (Fig. S2b). One balance was used to measure the volume exiting the cold and hot streams, and another balance was used to measure the pre-heating or pre-cooling volume from higher stages. From the mass measurement, the volume of water leaving the system was calculated using the density of water. The coefficients of performance as a refrigerator COP_r_, and heatpump COP_hp_, were calculated on a per-cycle basis using the mass of water *m*_w_, the specific heat capacity of water *c*_w_, the average temperature rise of the heated water from ambient *T*_h_, the average temperature drop of the cooled water from ambient *T*_c_, and the total non-recoverable work of the system *W*:

$${{\rm{COP}}}_{{\rm{r}}}=\frac{{Q}_{{\rm{c}}}}{W}=\frac{{m}_{{\rm{w}}}{c}_{{\rm{w}}}{T}_{{\rm{c}}}}{W}=\frac{{m}_{{\rm{w}}}{c}_{{\rm{w}}}({T}_{{\rm{amb}}}-{T}_{{\rm{unloading}},{\rm{avg}}})}{W}$$

$${{\rm{COP}}}_{{\rm{hp}}}=\frac{{Q}_{{\rm{h}}}}{W}=\frac{{m}_{{\rm{w}}}{c}_{{\rm{w}}}{T}_{{\rm{h}}}}{W}=\frac{{m}_{{\rm{w}}}{c}_{{\rm{w}}}({T}_{{\rm{loading}},{\rm{avg}}}-{T}_{{\rm{amb}}})}{W}$$

The total non-recoverable work was calculated as the active volume of NiTi for a particular stage *V*_s_ times the area enclosed in the stress-strain response for that particular stage, summed over the appropriate stages. The active volume is the volume of NiTi available for use as refrigerant (the NiTi beyond the tube fittings is used to fix the wires to the actuators/load cells and does not perform cooling or heating, see Fig. S7). As example, for the three stage system the non-recoverable work was calculated as:

$$W=\mathop{\sum }\limits_{{\rm{s}}=1}^{{\rm{s}}=3}\,{V}_{{\rm{s}}}\,\oint {\sigma }_{{\rm{s}}}\,d{\epsilon }_{{\rm{s}}}$$

This COP calculation assumes that the tension in the wire can be recovered upon unloading. Multistage systems are innately well-suited for work recovery; for example, this could be achieved by an even number of out-of-phase, opposing refrigeration systems^40^. We also assume that the work required to pump the water and the work to turn on and off the solenoid valves is negligible.

The specific heating ($\dot{q}$_h_) and cooling ($\dot{q}$_c_) power were obtained by dividing the heating or cooling capacity by the total mass of refrigerant used, *m*_NiTi_, and the total cycle time of the particular staging configuration, *τ*:

$${\dot{q}}_{{\rm{c}}}=\frac{{Q}_{{\rm{c}}}}{{m}_{{\rm{NiTi}}}\tau }=\frac{{m}_{{\rm{w}}}{c}_{{\rm{w}}}{T}_{{\rm{c}}}}{{m}_{{\rm{NiTi}}}\tau }$$

$${\dot{q}}_{{\rm{h}}}=\frac{{Q}_{{\rm{h}}}}{{m}_{{\rm{NiTi}}}\tau }=\frac{{m}_{{\rm{w}}}{c}_{{\rm{w}}}{T}_{{\rm{h}}}}{{m}_{{\rm{NiTi}}}\tau }$$

*T*_span_ was always measured using the average (not maximum or minimum) temperature of water leaving the system and is defined as *T*_h_ − *T*_c_. We sometimes used maximum or minimum temperatures to find optimal system parameters (Fig. 2). Δ*T*, Δ*T*_h_, and Δ*T*_c_ are analogous to *T*_span_, *T*_h_, and *T*_c_ except that the maximum and minimum temperatures were used instead of the average during flow.

The Carnot COP was calculated using the inlet water temperature (*T*_amb_) and the average hot (*T*_h_) and cold (*T*_c_) temperature changes of the water leaving the final stage:

$$\begin{array}{l}{{\rm{COP}}}_{{\rm{hp}},{\rm{carnot}}}=\frac{1}{1-{T}_{{\rm{L}}}/{T}_{{\rm{H}}}}\,{\rm{and}}\,{{\rm{COP}}}_{{\rm{r}},{\rm{carnot}}}=\frac{1}{{T}_{{\rm{H}}}/{T}_{{\rm{L}}}-1}\\ {\rm{where}}\,{T}_{{\rm{H}}}={T}_{{\rm{amb}}}+{T}_{{\rm{h}}}[{\rm{K}}]\,{\rm{and}}\,{T}_{{\rm{L}}}={T}_{{\rm{amb}}}-|{T}_{{\rm{c}}}|[{\rm{K}}]\end{array}$$

Also note that *T*_amb_ was not constant across all experiments, but varied between 21.5 to 23.5 °C.

### Nitinol training

All NiTi wires were trained (if unfamiliar with training, see ref. ^24^, for example) before use in any other experiments presented in this manuscript. To determine the number of cycles required for training, we measured the adiabatic temperature change of the NiTi when loaded and unloaded in air (Fig. S3). Elastocaloric response was stabilized after 100 cycles, so we performed training of all others wires for this number of cycles. Besides the wire used in the adiabatic temperature measurements, all wires were trained in water (Fig. S8) at the strain-rate of the perspective stage (Fig. 1c). We flowed a large amount of water over the wires after both loading and unloading so that the wires were at *T*_amb_ before the next loading/unloading step. After training we re-zeroed the strain on the wires to account for any non-recoverable strain incurred during training.

### Staging methodology and experimental replicates

Each of nine settings (three collection volumes and three strain values) was tested for four successive cycles for each of the five staging configurations. The first cycle was not used in data analysis in an attempt to avoid transient effects. Each data point in Figs. 2 and 3 represents the average value of the last three cycles for each setting. Single-stage, two-stage, and three-stage configurations were tested one after another without any modification to the system (only the loading/unloading of the first or second stage was omitted, where appropriate). The low-*A*_f_ wire was tested by replacing the high-*A*_f_ wire in the third stage with an equivalently sized wire, but otherwise no modifications were made (for example, the same tubing was used). The four-wire configuration was tested by replacing the first and second stage. Testing at each setting was limited to four cycles as the steady state response was reached within one or two cycles.

### Material fatigue

Our study did not closely monitor wire fatigue, but we can make rough estimates on the maximum number of loading/unloading cycles for the refrigerants. Only one wire (of seven total) failed during experiments conducted for the data presented in this manuscript, and we estimate that this occurred after at least 300 cycles (this wire was replaced with an equivalently sized one and was trained in the same manner). We estimate that some other wires were loaded/unloaded for at least 400 cycles and never failed. We suspect that fixing NiTi by opposing set screws was detrimental to the lifetime of the wires: large indentations were left in the wires at the location where the set screws were fixed (Fig. S1), and the wires that did fail always snapped at the location of the indentations. We strained the NiTi up to 4.2% on the observation that with no pre-heating or pre-cooling the superelastic plateau of the NiTi alloys ended at about that strain.

### NiTi materials

Two Nitinol alloys were used in this study. Except when stated otherwise, experiments used a higher-transformation-temperature (high-*A*_f_) superelastic NiTi alloy produced by Memry Corporation. Nickel-Titanium composition of this alloy was not available from the manufacturer. We also used a NiTi alloy produced by Fort Wayne Metals in select experiments (we call this the low-*A*_f_ wire because it has lower transition temperatures than the wire from Memry, see Fig. S4). The low-*A*_f_ wire is 56.2% Nickel (by weight) with the remainder Titanium. Individual trace elements are 0.02% or less (by weight). Both alloys were in wire form with diameter 1.27 mm. Given the geometry of the wires and Tygon tubing around the wires, the measured density of the NiTi wires (6.5 g/cm^3^), and the measured specific heat by DSC (about 0.55 J/g °C for both alloys), the total heat capacity of the NiTi was about 1.5 times that of the water surrounding it.

## Supplementary information

Supplementary information

Supplementary Movie
