# Supplementary material for: A multistage elastocaloric refrigerator and heat pump with 28 K temperature span
Source: Sci Rep. 2019 Dec 6;9:18532. doi: 10.1038/s41598-019-54411-8 (PMC6897911; doi:10.1038/s41598-019-54411-8)
Supplement: Supplementary file 1 — Supplementary information [file 41598_2019_54411_MOESM1_ESM.pdf]

Supplementary information

A multistage elastocaloric refrigerator and heat pump with 28 K temperature span

Ryan Snodgrass and David Erickson*

Sibley School of Mechanical and Aerospace Engineering, Cornell University, Ithaca, NY

*de54@cornell.edu

S1 Supplementary figures

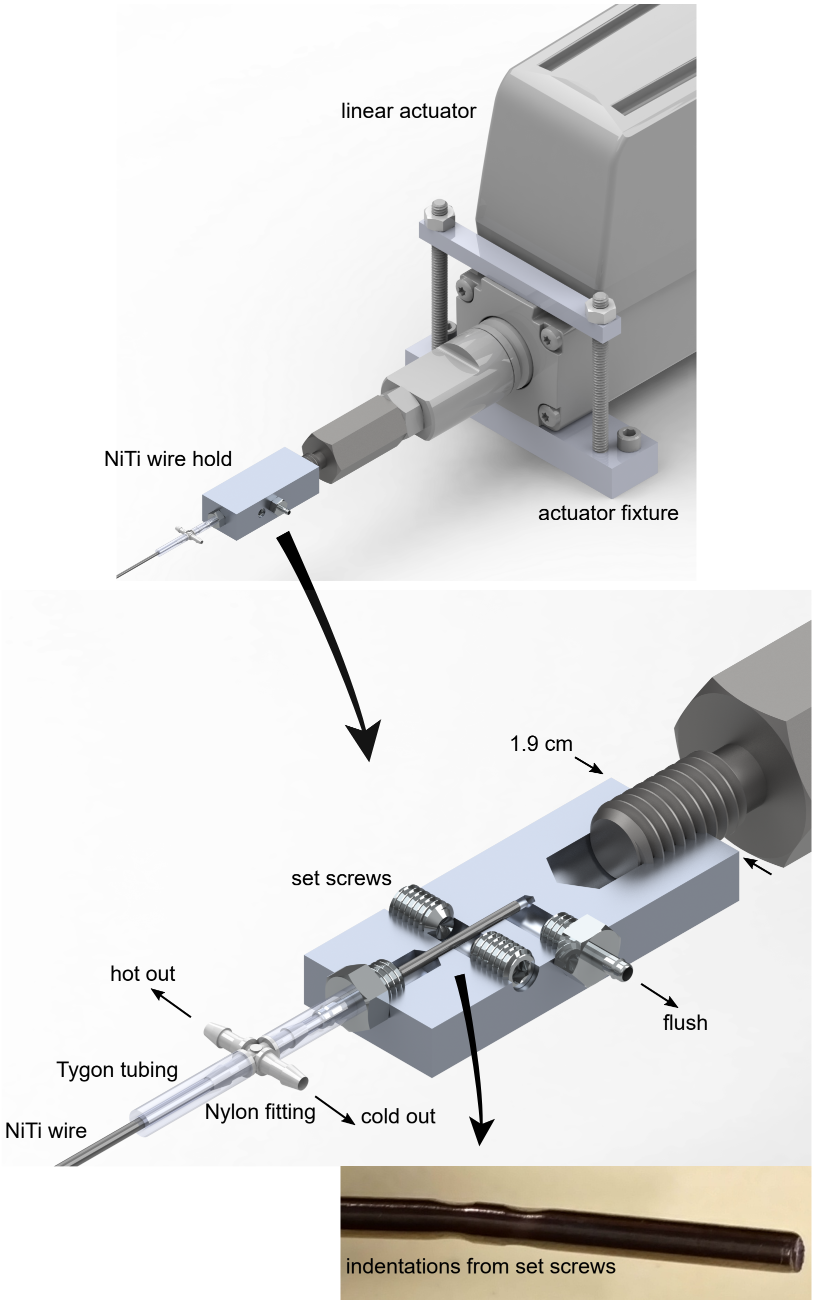

Fig. S1. Wire hold design. NiTi was fixed to the linear actuators and load cells using an aluminum part with two opposing set screws (thread 10-32 UNF). This part also has a flush port which was used to input water at the first stage and for exiting flush water at the third stage. Note that in staged configurations the hot and cold water does not travel through the aluminum pieces used to hold the wires but only travels through tubing and tube-fittings, otherwise a significant amount of heat would inadvertently be transferred to and from the holds. The Tygon tubing is quite flexible and was able to stretch with the wire during loading.

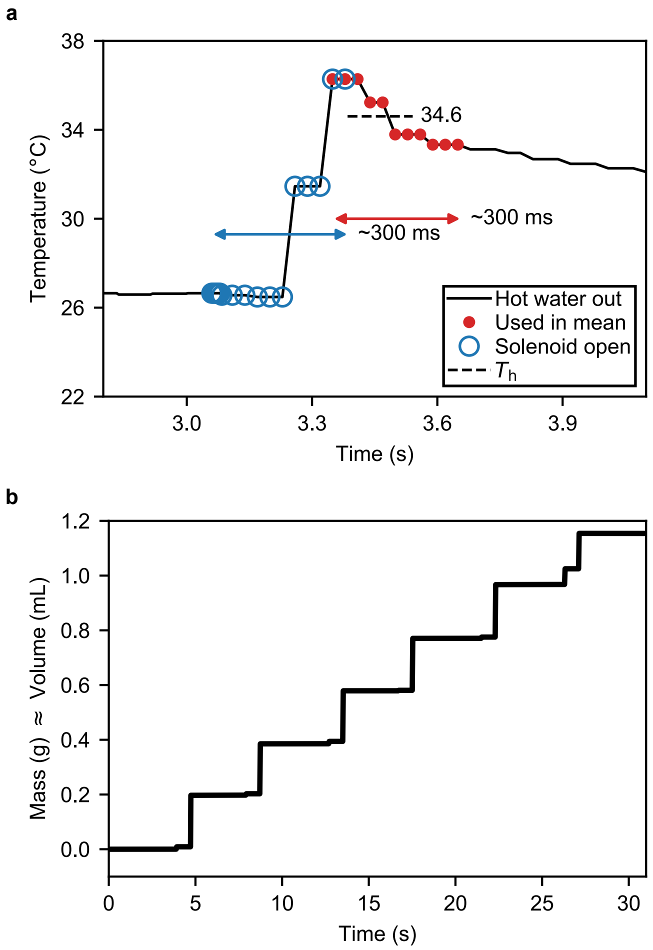

Fig. S2. Methods for measuring the temperature and mass of water leaving the refrigerator. a, Temperature measurement. A limitation of our system is a long cycle time (16 s for the three-stage configuration). Because the cycle time is so long and the volume of hot and cold water is so small (70 μL in some cases), we measured the temperature of the water leaving the system and not the temperature of heat sinks and sources; sinks/sources would have too much time to return to T_{amb} between cycles. T_{span} was calculated using average water temperature during the time that water was flowing out of the cold or hot streams, accounting for a delay of a few hundred milliseconds between the time that the microcontroller tried to open the solenoid valve and begin flow (first blue data point) and the time the thermocouple responded to the hot (or cold) water (first red data point). The delay can be attributed to the response time of the solenoid, the response time of the pump, and the heat capacity of the thermocouple. **b,** Mass measurement. The mass of water exiting the system was measured using analytical balances which communicated with the microcontroller via RS-232. Each step in the plot shows hot or cold water leaving the refrigerator; for example, this graph shows three complete load-unload cycles.

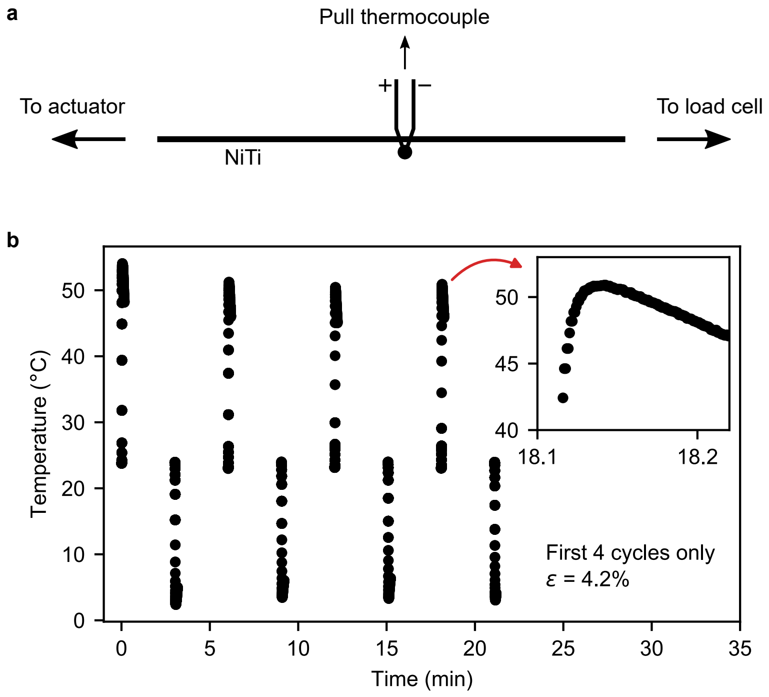

Fig. S3. Measurement of adiabatic temperature change. a, To ensure good contact with the refrigerant, we separated the ends of a K-type thermocouple (0.25 mm wire diameter) and straddled the NiTi inside the thermocouple, pulling slightly in a direction perpindicular to the NiTi. **b,** The first four cycles of loading/unloading of the same NiTi wire as shown in Fig. 1d. Data is discontinuous as temperature was only measured during loading/unloading and not during the entire return to T_{amb}.

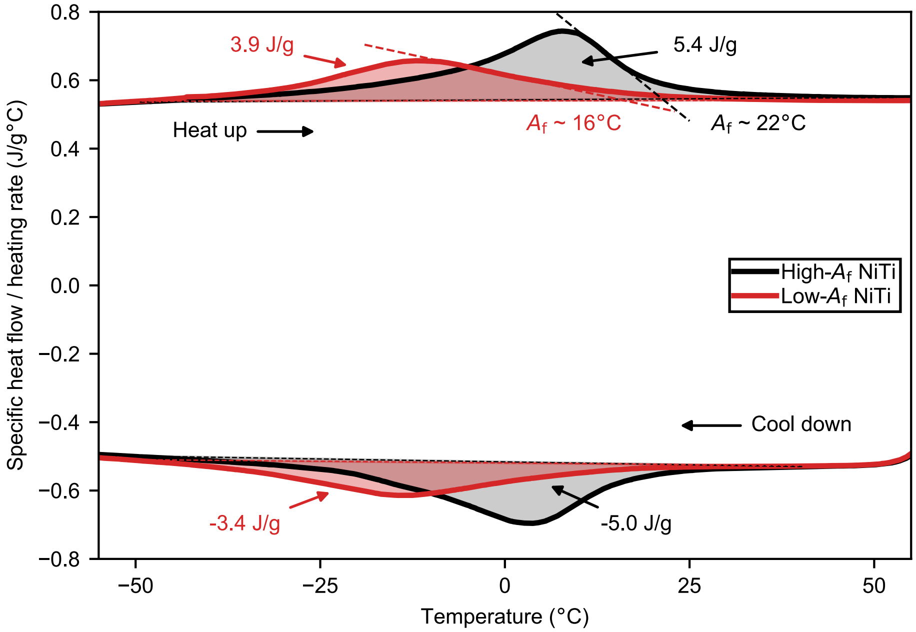

Fig. S4. Differential scanning calorimetry (DSC) of Nitinol alloys. DSC was performed on as-received wires using a TA Instruments Q1000 at a heating rate of 10 °C/min. The specific latent heats measured by DSC were lower than expected given the measured adiabatic temperature change (Fig. S3) and the reported latent heat of NiTi by other studies[1]. Still, this data is valuable to compare ΔH between the two alloys used in this study, as well as the difference in transition temperatures. Heat flow could not be measured at very low temperatures because liquid nitrogen cooling was not available.

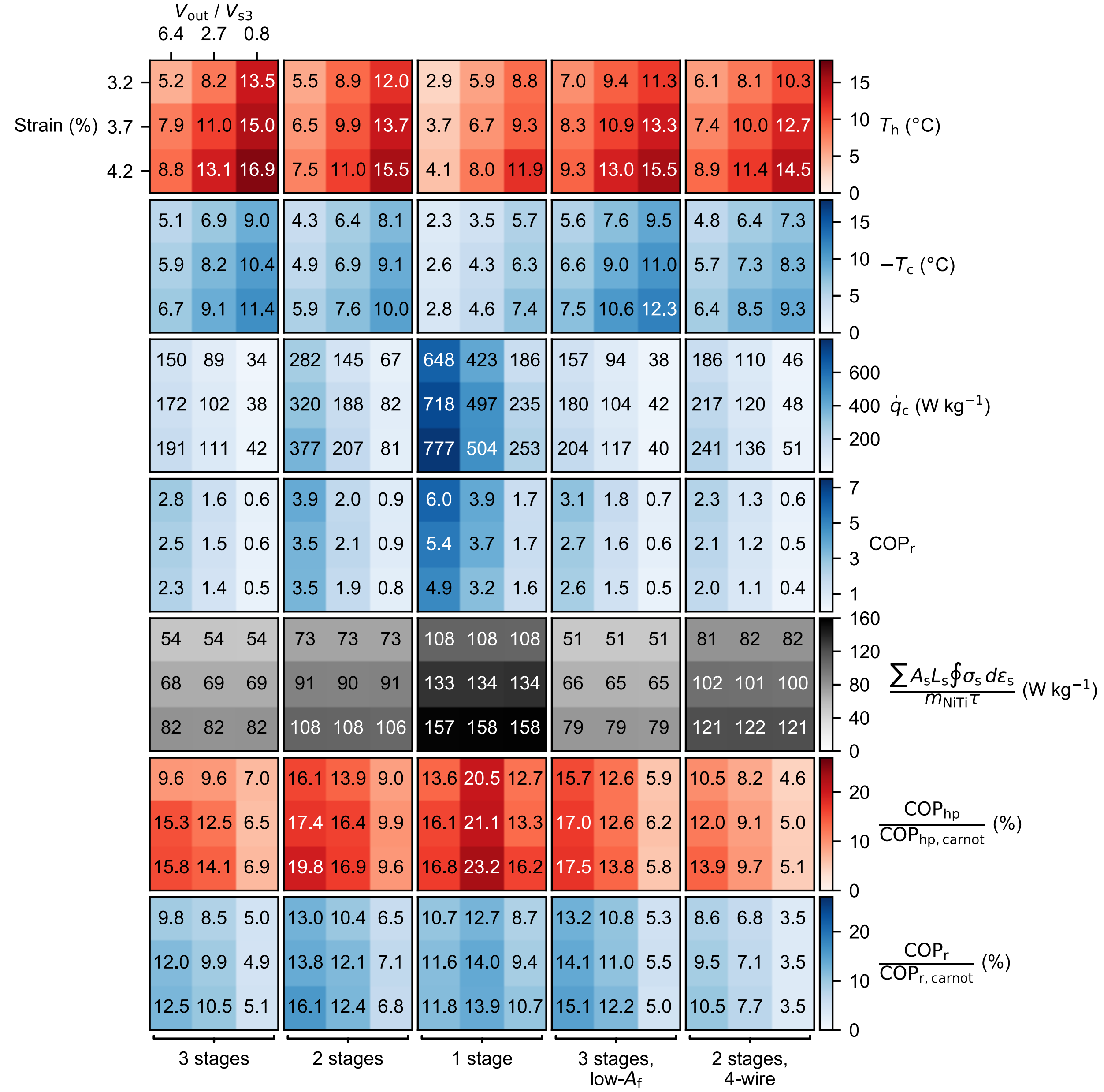

	3 stages	2 stages	1 stage	3 stages, low-A_f	2 stages, 4-wire
Refrigerant mass	5.44 g	3.25 g	1.10 g	5.45 g	5.49 g
Cycle time	16 s	12 s	8 s	16 s	11 s

Fig. S5. Supplementary measurements for each of five staging configurations. T_h, the average temperature rise of the hot water exiting the last stage of the heat pump (relative to the inlet). T_c, the average temperature drop of the cold water exiting the last stage of the refrigerator (relative to the inlet). $\dot{q}_c$, the specific cooling power. COP_r, the coefficient of performance as a refrigerator. The fifth row is the work input per cycle — calculated as the sum over all stages of the area enclosed by the stress-strain curve times the volume of active refrigerant — divided by the total refrigerant mass and cycle time, giving the input work per kg of refrigerant. The sixth and seventh rows are the COPs relative to Carnot (see Methods for calculation). V_{out}/V_{s3} is volume of hot or cold water collected normalized by the volume of water stored in the third stage (about 100 μL). Each nine-cell square shares the same strain and V_{out} layout as the top-left square, and each value in the heatmap is the average of three cycles. The total refrigerant mass and cycle time for each of the five staging configurations is shown at the bottom.

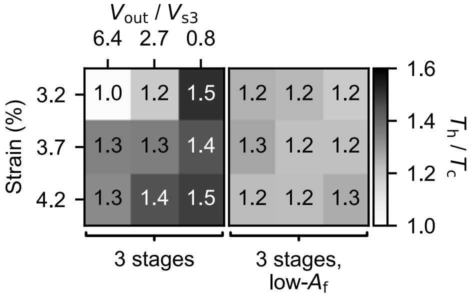

Fig. S6. Hot versus cold temperature change for different NiTi alloys. In a three-stage configuration, the ratio of hot-to-cold temperature change was generally higher for the high-A_f wire (left) than the low-A_f wire (right). This is possibly explained by pre-cooling effects, where the high-A_f wire experiences temperature-induced phase transformation before unloading. In Fig. 2c — a single-stage experiment with no pre-cooling — the ratio of the hot-to-cold temperature change for the high-A_f wire was close to 1.25 for sufficient flush volume, where in the three-stage experiments presented here the ratio was often between 1.4 and 1.5.

a

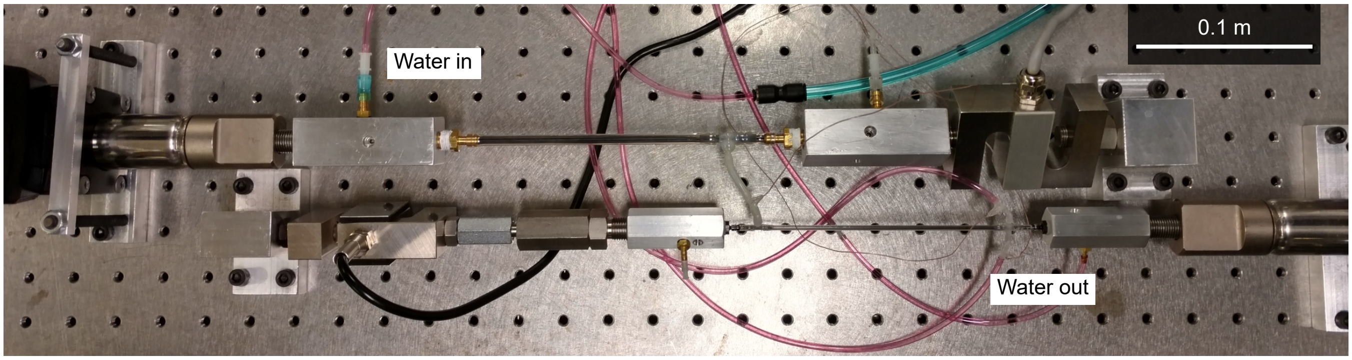

b

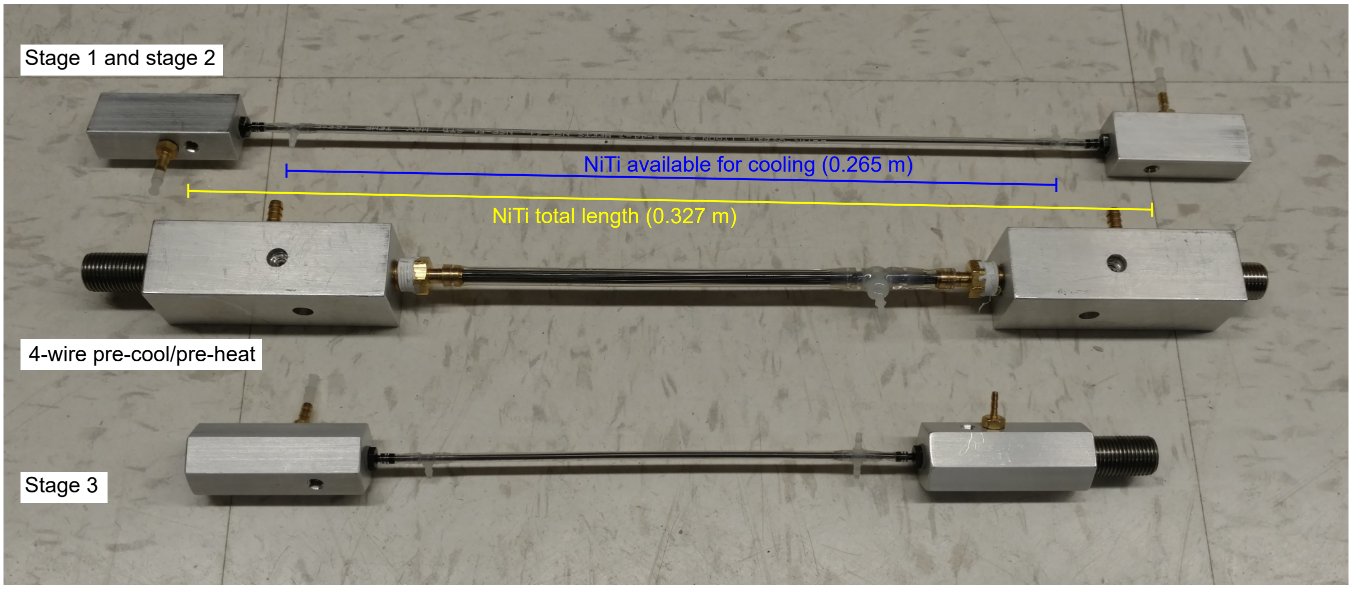

Fig. S7. Extra configurations for the staged refrigerator. a, An image of the system when operating with the 4-wire pre-cooling/pre-heating stage. The 4-wire stage is at the top and the last stage is at the bottom. The wire holds for the 4-wire stage are similar in design to those for the single-wire stages but larger and with 4 set screws. We would recommend other methods for holding multiple wires as our methodology was prone to rapid fatigue due to the excessive tightening required to prevent the wires from slipping. **b,** The system was designed to be relatively modular, as entire stages could be removed from the system by unscrewing the wire holds from the linear actuators and load cells without releasing the set screws that fix the NiTi. This improved workflow for the testing of a variety of staging configurations. This image also shows that only the portion of NiTi between tube fittings was available for cooling, although the length of NiTi fixed between set screws was longer.

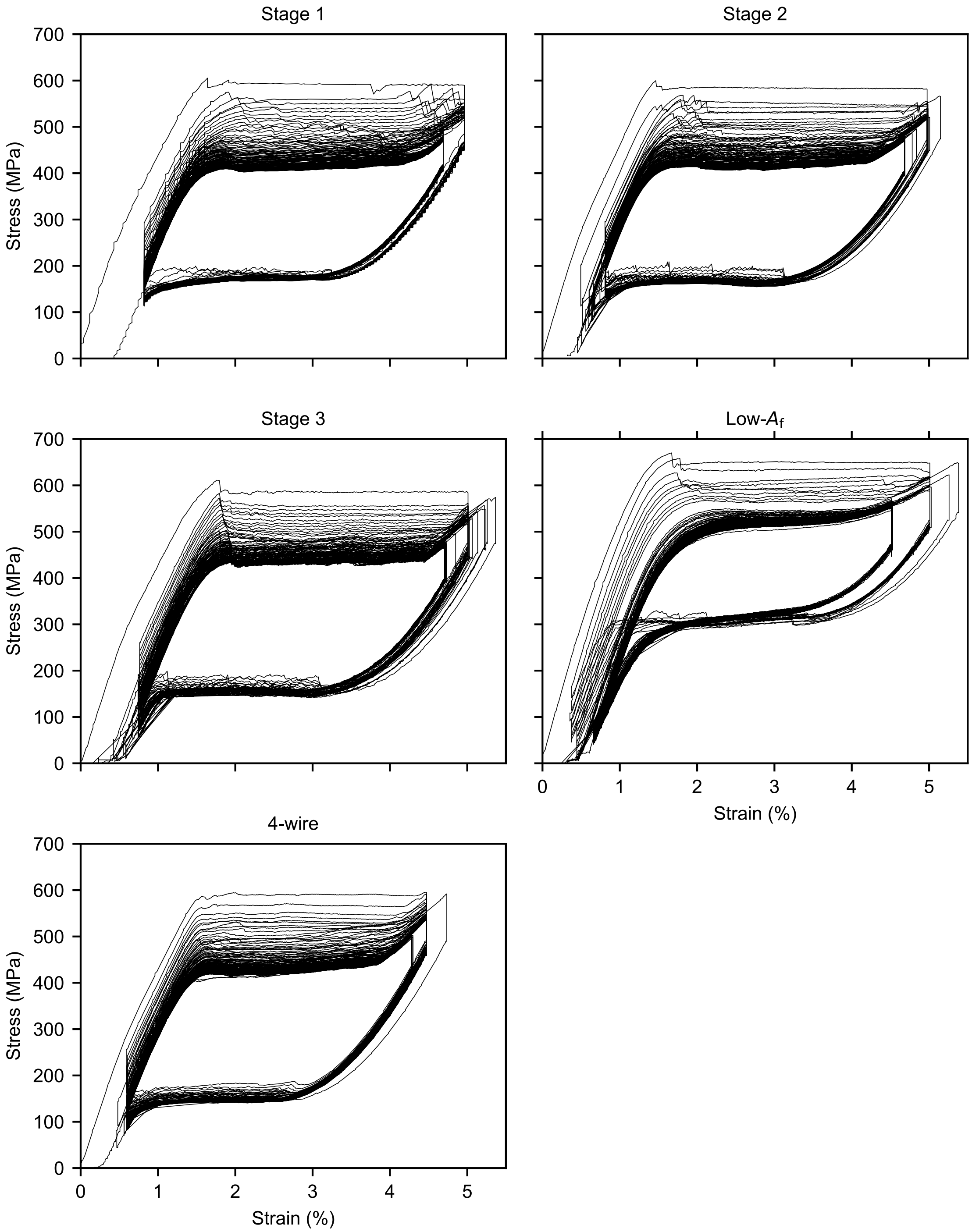

Fig. S8. Stabilization of the superelastic response (training). Each stage was trained for 100 cycles in water, starting with as-received wire(s). The wire was brought back to T_{amb} after loading and unloading by flowing a large amount of water over it. Training was performed at maximum strain rate by running each actuator at full speed (see Fig. 1c). The alloy used in stage 1, stage 2, stage 3, and the 4-wire pre-cooling stage was the high-A_{f} type. Strain exceeding 4.5% was unintentional and was an artifact of an imperfect braking mechanism.

References

1. Wieczorek, A. *et al.* Optimizing Ni–Ti-Based Shape Memory Alloys for Ferroic Cooling. *Functional Materials Letters* **10,** 1740001 (2016).
